# Comparison of microcoils and polyvinyl alcohol particles in selective microcatheter angioembolization of non variceal acute gastrointestinal hemorrhage

**DOI:** 10.12669/pjms.314.7240

**Published:** 2015

**Authors:** Tanveer-Ul-Haq, Muhammad Idris, Basit Salam, Waseem Akhtar, Yasir Jamil

**Affiliations:** 1 Dr. Tanveer-Ul-Haq, FRCR, Department of Radiology, Aga Khan University Hospital, Stadium Road, 74800 Karachi, Pakistan; 2 Dr. Muhammad Idris, FCPS, Department of Radiology, Aga Khan University Hospital, Stadium Road, 74800 Karachi, Pakistan; 3 Dr. Basit Salam, FCPS, Department of Radiology, Aga Khan University Hospital, Stadium Road, 74800 Karachi, Pakistan; 4 Dr. Waseem Akhtar, FCPS, Department of Radiology, Aga Khan University Hospital, Stadium Road, 74800 Karachi, Pakistan; 5 Dr. Yasir Jamil, FCPS, Department of Radiology, Aga Khan University Hospital, Stadium Road, 74800 Karachi, Pakistan

**Keywords:** Angio-embolization, Comparison, Gastrointestinal haemorrhage. Microcoils, Polyvinyl alcohol particles

## Abstract

**Objectives:**

To compare the efficacy of polyvinyl alcohol (PVA) particles with microcoils in angiembolisation of non variceal acute gastrointestinal haemorrhage.

**Methods:**

This is a retrospective cross-sectional study of patients who underwent transcatheter angioembolization from January, 1995 to December, 2013 at Aga Khan University Hospital, Karachi. Patients were divided into two groups on basis of use of either microcoils or PVA particles and compared in terms of technical success, clinical success, re-bleeding and ischemic complication rates. Chi (χ^2^) square and Fisher’s exact tests were applied and a P-value of less than 0.05 was considered statistically significant.

**Results:**

Fifty seven patients underwent angioembolization. Microcoil and PVA particles embolization was performed in 63% (36/57) and 35% (20/57) cases respectively. Technical success was achieved in all cases (100%). Clinical success rate was higher in microcoils group (92%) than PVA particles group (75%) with statistically significant P value (p=0.048). Ischemic complication was seen in one case (3%) in the microcoil group, while no such complications were seen in the PVA particles group.

**Conclusion:**

In angioembolization of non variceal acute gastrointestinal haemorrhage microcoils are better than Polyvinyl alcohol particles with higher clinical success and lower re-bleed rates.

## INTRODUCTION

Non-variceal acute lower gastrointestinal haemorrhage (NVAGIH) is a life threatening abdominal emergency. Anatomically, gastrointestinal haemorrhage has been divided into upper and lower gastrointestinal bleeding by the ligament of Treitz.^1^ Lower gastrointestinal (LGI) haemorrhage has an incidence of 20.5 per 100,000 patients with a mortality rate of about 2-5%.^2^,^3^ Most cases of gastrointestinal haemorrhage are controlled with supportive measures, but, a significant number of patients require further interventions, which often involve collaborative efforts between gastroenterologists, surgeons and interventional radiologists. Among the available treatment modalities, endoscopy is the modality of first choice.^4^,^5^ However, in some emergency situations, presence of faecal matter and blood clots can obscure the source of the haemorrhage making endoscopy inconclusive.^6^ Surgery offers curative and definitive treatment, but, it is associated with significant morbidity and mortality ranging from 10-15%.^7^ Particularly, in patients with severe co-morbidities, this mortality rate reaches up to 30%.^8^

Catheter angioembolization is becoming a popular and viable option for the management of patients with NVAGIH. Multiple embolic materials are available like detachable fibre coils, platinum coils, detachable balloons, particles and gel foam.^9^,^10^ Among available embolic materials, microcoils are easy to use as compared to polyvinyl alcohol (PVA) particles. Extensive literature has been published on the technical success, clinical success and complications rates of catheter angioembolization, but, only a few studies have compared various embolic agents in terms of their individual performance. In this study, we share our 18-year experience of catheter angioembolization in NVAGIH with special focus on efficacy and safety of PVA particles vis-á-vis traditional microcoils.

## METHODS

This retrospective study was approved by the institutional ethical review board and requirement of informed consent was waived. A total of 167 patients with NVAGIH underwent catheter angiography at the radiology department of our institution during a period of 18 years (January, 1995 to December, 2013). Out of these, angiography did not reveal any active contrast extravasation in 106 patients (63%). These patients were managed conservatively and subsequently discharged from our hospital in stable condition. In 61 patients (37%), there was active extravasation of contrast on angiography and they underwent angioembolization using microcoils, PVA particles or a combination of these. Three patients from the microcoil and one patient from the PVA particles group were excluded owing to incomplete medical records and/or lost to follow-up. Incomplete records was defined as unavailability of daily progress notes, physical examination findings and/or laboratory results, especially coagulation profile, platelet count and haemoglobin level. The presence of all of these clinical parameters along with complete laboratory results, daily progress notes before and after embolization, surgical and endoscopy notes (if any) were necessary to label a medical record as complete. Radiological data was retrieved from our Radiological Information System (RIS) and clinical data was obtained from the medical records of our hospital. Patients included in this study were categorized on the basis of the embolization material used into two groups i.e. microcoil group (patients treated with microcoils only) and PVA particles group (patients treated with PVA particles only). Both groups were compared in terms of patient demographics, presence of co-morbidities, technical success, clinical success, re-bleeding and procedure-related bowel ischemia. Technical success was defined as complete cessation of bleeding achieved at the end of the angioembolization procedure as demonstrated by final angiogram. Clinical success was defined as complete cessation of bleeding with angioembolization followed by stability in haemodynamics and no re-bleeding within two weeks post-procedure. Re-bleeding was assessed by pursuing clinical parameters (clearing of aspirate from nasogastric tube, no blood per rectum and assessment of haematologic and haemodynamic parameters). Procedure-related bowel ischemia was diagnosed by clinical, endoscopic and surgical findings.

### Embolization technique

**Fig. 1:**
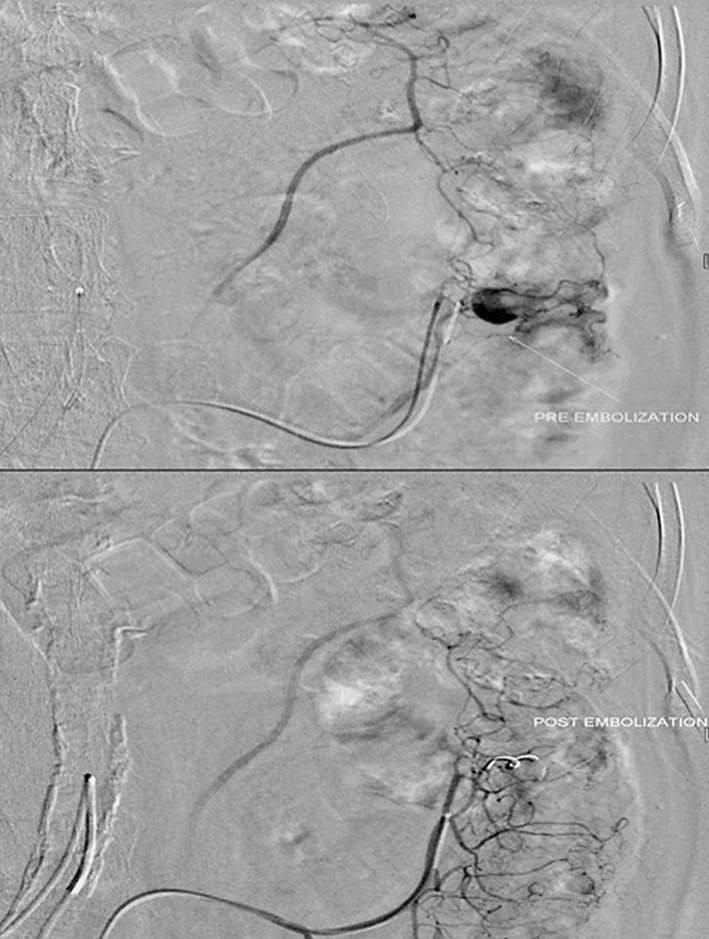
Micro coil angioembolisation. Digital subtraction angiography image showing catheter tip in distal branch of left colic artery showing active bleed marked by long white arrow. Subsequently angioembolisation performed by placement of platinum microcoil and complete cessation of bleeding achieved.

**Fig. 2:**
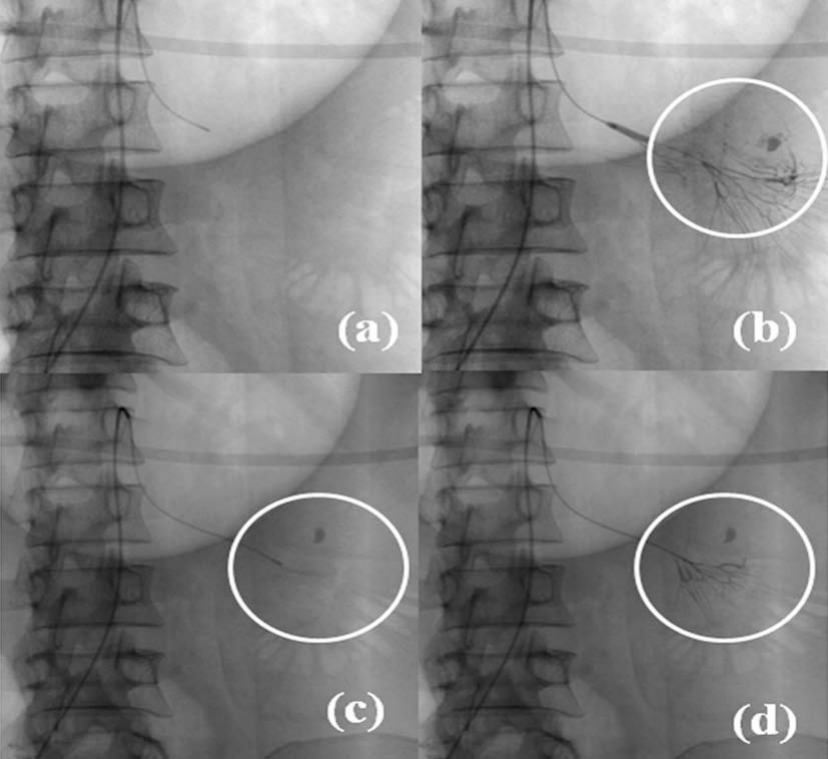
Poly vinyl alcohol particle angioembolisation. a): Digital subtraction angiography image showing catheter tip in one of the jejunal branch of superior mesenteric artery. b): Subsequent angiogram showed active bleeding in one of the distal jejunal branch of superior mesenteric artery marked by circle. c): Superselective cannulation of bleeding vessel by further manipulation of microcatheter. d) Post particle embolisation angiogram showing complete cessation of bleeding marked by circle (Note: compare with Fig.2b).

Diagnostic angiography was performed in all patients by standard transfemoral catheterization technique using a 5-French vascular access sheath inserted under ultrasound and fluoroscopic guidance and a 4-French Cobra or Simmon catheter (Cordis, Johnson and Johnson, FL, USA) over a 0.035” Terumo Radifocus guide wire (Terumo medical corporation, NJ, USA). First, selective angiography of celiac, superior mesenteric and inferior mesenteric arteries was done depending on the findings of endoscopy and/or red blood cell (RBC)-tagged scintigraphy. Once the site of bleeding was identified, superselective angiography was performed using a 2.9-French microcatheter (Progreat, Terumo, Japan), which was inserted co-axially through the already placed 4-Fr catheter. Extravasation of contrast media and/or the presence of arterial pseudo-aneurysm indicated bleeding. Microcatheter was then manipulated to reach the vasa recta and angioembolization was performed by using either microcoils (size range: 0.015”-0.018”; Balt, Extrusion, France) [Fig.1] or PVA particles (size range: 250-350 μm; Boston scientific, Natick, MA, USA) [Fig.2] or both.

### Data analysis

Data analysis was performed on SPSS software version 14.0. Frequency and percentages were computed for categorical variables, while means and standard deviations were calculated for continuous variables. Pearson Chi-Square (χ^2^) and Fisher exact tests were used to compare technical success, clinical success, re-bleeding and procedure-related bowel ischemia among both groups. A *p*-value of less than 0.05 was considered statistically significant. Multivariate analysis of various factors affecting clinical success was also performed.

## RESULTS

A total of 57 patients were included in the final analysis. Of these, 63% (36/57) were treated with microcoils, 35% (20/57) with PVA particles and 2% (1/57) with both materials. In the microcoil group, 75% (27/36) were male (age range: 21-87 years) and 25% (9/36) were female (age range: 18-77 years), while in the PVA particles group, 70% (14/20) were male (age range: 9-75 years) and 30% (6/20) were female (age range: 15-64 years). In the microcoil group, 50% (18/36) had bleeding per rectum, 47% (17/36) had melena and 3% (1/36) presented with haematemesis. In the PVA particles group, 70% (14/20) patients had bleeding per rectum and 30% (6/20) had melena. Coagulopathy was the most common co-morbid condition in both groups. Other co-morbidities included uraemia, chronic liver disease, hypertension and diabetes mellitus. (Table-I)

**Table-I:** Patients Base Line Data. Multivariate analysis. Comparison of microcoils and PVA particles.

Variable	Parameters	Microcoils (36)	PVA particles (20)	P-value (95% CI upper, lower)	Adjusted P-value (95%CI upper, lower)
Gender of patients	Males	27 (75%)	14(70%)	0.332 (.540, 6.202)	0.798 (.2276.879)
	Females	9 (25%)	6 (30%)		
Major comorbids	Chronic liver disease	7 (12%)	5 (17%)	0.442(.273,19.460)	0.841 (.10116.815)
	Coagulopathy	24 (41%)	7 (24%)	0.226 (.154, 1.556)	0.483 (.0693,.543)
Major symptoms	Bleeding per rectum	18 (50%)	12 (60%)	0.608 (.231, 2.357)	0.862 (.253,3.157)
	Malena	17 (47%)	6 (30%)	0.267 (.154, 1.556)	0.493 (.0693,.543)
Blood transfusion	Transfusion done	28(78%)	13 (65%)	0.907(.262, 3.278)	0.665(.240,9.358)

In the microcoil group, 78% (28/36) patients underwent blood transfusions as compared to the PVA particles group, where only 65% (13/20) patients underwent blood transfusions. As part of initial work-up and treatment, endoscopy was performed in 56% (20/36) and 60% (12/20) of patients in the microcoil and PVA particles groups respectively. In these patients, endoscopic methods of haemostasis remained unsuccessful and they were subsequently referred for catheter angiography and embolization. RBC tagged scintigraphy was performed in 39% (14/36) and 5% (1/20) patients in the microcoil and the PVA particles groups respectively. The most common site of contrast extravasation was the caecum in both groups i.e. 58% (21/36) and 70% (14/20) in the microcoil and PVA particles groups respectively. The most commonly embolized vessel was the ileocolic artery and its branches i.e. 58% (21/36) and 70% (14/20) in the microcoil and the PVA particles groups respectively. Mean follow-up was 19.1 days (range: 13-35 days) in the microcoil group, while in the PVA particles group, it was 16 days (range: 13-25 days).

Technical success was achieved in all patients of both groups. Clinical success rate was higher with microcoils (33/36 = 92%) than with PVA particles (15/20 = 75%) [*p*=0.048]. Clinical success was also achieved in one patient who underwent angioembolization with a combination of both microcoils and PVA particles. (Table-II) Patients undergoing angioembolization with microcoils had a re-bleeding rate of 8% (3/36), while it was 25% (5/20) for patients angioembolized with PVA particles (*p*=0.048).

**Table-II:** Angioembolisation performance. Comparison of microcoils and PVA particles

	Microcoils (36)	PVA (20)	P value
Technical success	100% (36/36)	100% (20/20)	NA
Clinical success	94% (34/36)	75% (15/20)	0.048
Rebleed rate	6% (2/36)	25% (5/20)	0.048
Bowel Ischemia	3% (1/36)	0% (0/20)	0.643

*Note:* Numbers given in parenthesis are patient numbers.

Procedure-related bowel ischemia was encountered in only one patient undergoing microcoil embolization. This particular elderly male patient was a known case of diabetes mellitus, hypertension and peptic ulcer disease. He had recurrent episodes of LGI bleeding in the past, which were treated with coil and PVA embolization in the region of ascending colon. He also had a previous history of laparotomy for repair of ileal perforation. This time he had presented with bleeding per rectum. On endoscopy, a bleeding focus was found in the ascending colon, which was refractory to endoscopic treatment and he was subsequently referred for angiographic embolization. On angiography, there was active extravasation in the territory of the right colic artery, which was successfully embolized by placing multiple microcoils (size: 0.015”; Balt, Extrusion, France). On second post-procedure day, he developed abdominal pain along with a fever of 38°C and tachycardia (pulse rate of 109/min). On clinical examination, his abdomen was tense and tender. His white cell count was also elevated (12 x 10^3^ μL). Exploratory laparotomy was performed, which revealed an ischemic perforation in the ascending colon, for which right hemicolectomy was performed. Post-operatively, he remained well and was subsequently discharged thereafter.

## DISCUSSION

Superselective transcatheter angioembolization has become a safe and effective therapeutic option for patients with LGI bleed. This procedure can be performed by using temporary as well as permanent embolic agents.^9^,^10^ Many studies have been published regarding the technical success, clinical success, re-bleeding rate, procedure-related bowel ischemia and mortality rate of angioembolization performed with various embolic agents. However, most of these were based on a combined analysis of various embolic agents. To the best of our knowledge, a comparative analysis between various embolic agents in terms of their efficacy and safety has not been published before. We conducted a retrospective comparative analysis in our set of patients who underwent angioembolization using either microcoils, PVA particles or both.

In our study, technical success was achieved in all cases of LGI bleed with both microcoils and PVA particles. This finding is in line with multiple studies reported in the literature on GI transcatheter embolization.^10^-^25^ In studies conducted on LGI bleed and achieving 100% technical success, Funaki et al.^13^ and Gillespie et al.^23^ used microcoils, while Guy et al.^11^ used PVA particles. In the rest of the studies,^12^,^14^,^17^ more than one embolic agent was employed and comparative analysis was not done. Many other studies have reported variable technical success rates ranging from 52% to 99%.^17^-^24^

In our study, clinical success ranged from 75% (for PVA particles) to 92% (for microcoils) and, interestingly, microcoils appeared to have a slightly higher clinical success rate than PVA particles. Heterogeneity in the granulometric size range of PVA particles may result in the formation of small aggregates within the microcatheter, thereby increasing the theoretic risk of injecting very small PVA particles through the microcatheter. However, it is also important to note here that the difference in the clinical success rates between the two groups was only marginal (*p*=0.048) and this difference may be a mere consequence of selection bias due to the discrepancy in the number of patients between the two groups (n=20 for the PVA particles group versus n=36 for the microcoils group). Among the studies published in the literature, Gordan et al. reported a clinical success rate of 100% by using microcoils in eight patients,^18^ while Waugh et al. reported the same for PVA particles in nineteen patients.^19^ Mensel et al. used microcoils for embolization of both upper and LGI bleeding in acute settings and achieved technical and clinical success rates of 88.6% and 56.8% respectively.^24^ Re-bleeding in our patients was significantly higher with PVA particles (25%) than with microcoils (8%). However this difference also remained borderline in terms of its statistical significance (*p*=0.048). In the published literature, re-bleeding rate was 0% in the LGI bleed series reported by Gordon et al.^18^ and d’Othee et al.^15^, both of whom used microcoils. Similar results were reported by Bandi et al.^20^, though they used more than one embolic agent.

In our study, there was only one case of procedure-related bowel ischemia secondary to microcoil embolization of the right colic artery. This elderly frail patient with multiple co-morbidities had been angioembolized in the past in the same vascular territory and a repeat angioembolization in the same area blocked all collateral vessels resulting in ischemia of the involved segment. In multiple studies done on angioembolization for LGI haemorrhage, no procedure-related bowel ischemia was seen with either microcoils or PVA particles.^11^,^12^,^18^,^21^

### Limitations of the study

These must be borne in mind before drawing any conclusions. Owing to the retrospective study design, sample size was not calculated and the number of patients in the two groups was not equal. Furthermore, several interventional radiologists were involved in the angioembolization procedures and this could have potentially influenced the clinical success rates among the two groups. Patients included in our study had a relatively short follow-up period (19.6 days on average) and some cases of re-bleeding might have been missed. Furthermore, due to the short follow-up period, long term safety of the procedures could not be assessed.

Despite the limitations of our study, we have provided a retrospective comparative analysis between microcoils and PVA particles as embolic agents for angioembolization in patients with LGI bleeding. The results of our study demonstrate the feasibility and safety of both embolic agents for angioembolization of patients with NVAGIH. Microcoils, being easier to use, can be employed in cases where the microcatheter can easily reach the vasa recta. However, in technically difficult cases where the microcatheter tip cannot be manipulated to reach as distal as the vasa recta, PVA particles may be a better option. The difference in the clinical success and re-bleeding rates noted between microcoils and PVA particles in our study is intriguing and warrants further validation through prospective controlled studies.

## CONCLUSION

In angioembolization of NVAGIH, PVA particles are both safe and effective option. However, microcoils have higher clinical success and lower re-bleeding rates than PVA particles.
